# Comparison of Three Wayfinding Assessment Tools

**DOI:** 10.1093/geroni/igaa057.1415

**Published:** 2020-12-16

**Authors:** Margaret Calkins, Rebecca Davis

**Affiliations:** 1 IDEAS, Cleveland Hts., Ohio, United States; 2 Grand Valley State University, Grand Rapids, Michigan, United States

## Abstract

An early, problematic symptom of aging, exacerbated by cognitive problems such as Alzheimer’s disease, is difficulty finding one’s way. Long term care (LTC) communities are especially challenging for wayfinding. This study compares three different tools to evaluate spatial complexity within eight LTC communities. The tools included computation of space syntax integration values using axial lines to determine how connected wayfinding routes are within the community. The Wayfinding Checklist tool (revised) rates the quality of signage, complexity of a building floor plan, and use of decorative elements. The Calkins Route Assessment tool evaluates route complexity using both plan and vertical visual field information (e.g. presence, location, clarity of signage and cues). The results of the analysis showed that the buildings varied with respect to integration, with some very well integrated floorplans and routes, and some that were very complex, with multiple jogs, turns, and limited integration. The Wayfinding Checklist echoed the variety of complexity of floorplans, but lowered scores for poor existence and design of signage and mostly substandard lighting for visibility. The Calkins Route Assessment tool added greater specificity to the space syntax analysis and Wayfinding Checklist, showing how visual elements (versus just visual access) can compensate for plans with low-integration. These study findings indicate that each tool was valuable for measuring wayfinding complexity, but measured different aspects of building and route complexity. Tools like these can be used to identify challenging wayfinding routes and buildings so that designers and clinicians can improve the overall design for wayfinding.

